# Academic Management in Uncertain Times: Shifting and Expanding the Focus of Cognitive Load Theory During COVID-19 Pandemic Education

**DOI:** 10.3389/fpsyg.2022.647904

**Published:** 2022-06-17

**Authors:** Douglas J. Gould, Kara Sawarynski, Changiz Mohiyeddini

**Affiliations:** Department of Foundational Medical Studies, Oakland University William Beaumont School of Medicine, Rochester, MI, United States

**Keywords:** COVID-19, pandemic, cognitive load theory, intrinsic load, extraneous load, academic management

## Abstract

Globally, the COVID-19 pandemic has forced medical education toward more “online education” approaches, causing specific implications to arise for medical educators and learners. Considering an unprecedented and highly threatening, constrained, and confusing social and educational environment caused by the COVID-19 pandemic, we decided to shift the traditional focus of the Cognitive Load Theory (CLT) from students to instructors. In this process, we considered recent suggestions to acknowledge the psychological environment in which learning happens. According to this fundamental fact, “Learning and instructional procedures do not occur in a situational vacuum.” Following this assertion, we adapted and implemented principles of CLT to reduce the extraneous load for our faculty to facilitate continued scholarly activity and support the overall wellbeing of our faculty during these trying times. The adoption of these principles enabled our team to cultivate attitudes and skills across multiple domains, such as online presentation technologies, implementing and maintaining a “classroom atmosphere” in a virtual environment, encouraging discussion among large online groups of students, facilitating group work, providing virtual office hours, and proactively planning for subsequent sessions.

## Introduction

Globally, the COVID-19 pandemic has forced higher education toward more “online education” approaches, causing specific implications to arise for educators and learners. As medical education faculty working during the COVID-19 pandemic from the metro Detroit area, one of the most severely affected areas in the United States, our department needed to quickly develop mechanisms to move entirely online within a few short days. Medical schools have a professional obligation and duty to deliver high-quality education that enables medical students to graduate and to provide essential health care to society.

The Foundational Medical Studies department is a large and multi-disciplinary basic and social science group. As such, we faced a host of challenges to maintain the quality of our teaching and academic community, while shifting abruptly to an online world. Such an abrupt shift, from traditional face-to-face interactions to online, requires cultivation of attitudes and skills across multiple domains among instructors (Todd et al., 2017; El Sayed and Abdelmonem, 2019). These domains include using online presentation technologies, cultivating and maintaining a “class room atmosphere” in our teaching efforts in a virtual environment, encouraging discussions among large online groups of students and faculty, facilitating group work, providing virtual office hours, and proactively planning for the following academic semesters. Acquiring these skills are essential to fostering a positive learning experience for students and colleagues in an unprecedented and highly threatening, constrained, and confusing social and educational environment caused by the COVID-19 pandemic (The Lancet, 2020; Tuttle, 2020).

## Challenges of an Abrupt Shift in Teaching Approaches of the Instructors

It seemed plausible to us to assume that a rapid pivot from face-to-face teaching, supervision, and mentoring to completely online instruction would cause many unknown challenges for the instructors. The complexity and quantity of required skills and knowledge alone would generate a significant cognitive load for the instructors, challenge the instructors’ learning and information processing capabilities which, in return, could increase errors, frustration, and harm both the teaching performance and wellbeing of the instructors (Haji et al., 2015; Sewell et al., 2019). In addition, the anticipated negative impacts of the COVID-19 pandemic suggested a threat to our culture of caring and empathy, open communication, collaboration, and collegiality among instructors who were suddenly cut off from their daily work and collaborative routines (Van Mol et al., 2015; Scoglio et al., 2018). Hence, the main priority of the departmental leadership was to manage this abrupt shift to online-only teaching, supervision, and mentoring while reducing the amount of cognitive load for instructors.

Online education is not novel (Castro and Tumibay, 2021) and online instruction is ubiquitous in medical education (Jiang et al., 2021). However, COVID-19 imposed an unprecedented urgency on academic institutions to pivot to online instruction without any lead time to plan and train instructors. Developing technology-based online instruction and teaching material is typically a time consuming task that requires careful planning and extensive training (Robinia and Anderson, 2010; Hampton et al., 2020; Wilson et al., 2021). Furthermore, it requires administrative and institutional support to implement and evaluate (Chiasson et al., 2015 Richter and Idleman, 2017). An additional challenge was that, prior to COVID-19, many instructors did not have prior experience with online education and most courses were not equipped with materials suitable for online instruction. This issue was highlighted by an Inside Higher Ed’s Survey in 2018 that revealed only 42% of the faculty polled had taught online courses (Jaschik and Lederman, 2018). Therefore, in this paper, we aim to demonstrate that in the absence of any prior experience and evidence-based recommendations for how to pivot the entire curriculum of a medical school to online instruction, psychological theories can provide guidance to develop and implement such a pivot. We aim to corroborate that the application of cognitive theories of learning, such as Cognitive Load Theory (CLT; Sweller, 1988, 2010), can help to further advance the teaching quality of instructors in times of the COVID-19 pandemic and to identify the diverse challenges instructors face. The identification and prevention of potential sources of a cognitive overload for faculty, specifically during times of uncertainty, such as the COVID-19 pandemic, seem critical to supporting the teaching performance and wellbeing of instructors. Previous research has demonstrated that instructors who feel challenged report higher levels of stress and burnout (Alschuler, 1980; Ochiai, 2003), display lower levels of teaching motivation, implement fewer effective teaching strategies, provide unclear instructions, and demonstrate ineffective classroom management to create a supportive and stimulating classroom climate for their students (Skaalvik and Skaalvik, 2021).

Therefore, we decided to shift the focus of the traditional CLT, which has been almost exclusively toward students’ learning (Sweller, 1994, 2010) and apply CLT to enhance instructors’ learning capabilities, to enable them to provide high-quality online education to our students (Naismith et al., 2015). Our theory-driven approach provides a novel avenue to enhance the quality of online instruction along with the purely technical/technology-based teaching aids, such as online web-based tools (Wilson et al., 2021), narrated PowerPoint presentations (Hampton et al., 2017), recorded lectures (Broussard and Wilson, 2018), use of videos (Forbes et al., 2016), assistance from an instructional designer (Wilson et al., 2021), blogs (Kaup et al., 2020), flipped classroom (Flugelman et al., 2021), and online case studies (Jeppesen et al., 2017).

## Cognitive Load Theory

Following Atkinson and Shiffrin’s model of human memory (Atkinson and Shiffrin, 1968), CLT assumes that a human’s learning system is comprised of three subsystems of memory (sensory, working, and long-term memory; Issa et al., 2011).

Sensory memory (SM) has no-known limitation to perceive visual and auditory information. However, it can retain information for only a very few seconds (Mayer, 2010; Eriksson et al., 2015). For that information to enter the domain of the Working Memory (WM), it must rise to the level of awareness. Hence, most of the information entering SM vanishes and only a small portion of the sensory information enters the domain of WM (Simons and Chabris, 1999; Brady et al., 2016). In contrast to SM and long-term memory (LTM), WM is finite (Miller, 1994), and its main function is parceling information by rearranging and reorganizing them into packages to facilitate efficient storage in LTM (Öğmen and Herzog, 2016; Wingfield, 2016). LTM has theoretically an infinite capacity and has no limitations in terms of duration and volume of information that it can store (Witt et al., 2019). However, like any well-managed library, LTM entitles a network of classification and categorization of information built of meaningful connections and route maps of cognitive strategies called schemas (in the context of medical education referred to as illness scripts, Custers, 2015; Whitehead et al., 2016; Gavinski et al., 2019). Cognitive schemas are prototypes of learning and problem-solving strategies that are created and refined over the course of ontological learning history (Disner et al., 2011; Bartels et al., 2017; McArthur et al., 2019).

In processing new information, WM retrieves and reactivates matching cognitive schemas from LTM to encode the information (Squire and Dede, 2015; Norris, 2017). The more often a schema is activated, modified, and successfully applied, the faster new information is encoded, parceled, and forwarded to and from LTM (Sporer, 2016; Fernández et al., 2017). Therefore, the process of schema automation and schema construction are, according to CLT, the key steps in the process of learning (Van Merriënboer and Sweller, 2010).

CLT assumes that learners encounter three sorts of cognitive loads in the process of learning (Sweller, 2010): intrinsic, extraneous, and germane cognitive load.

Intrinsic load is associated with the content of the material and processing of the information to comprehend the material (Young et al., 2016; Klepsch et al., 2017; Jordan et al., 2020). Put simply, intrinsic load relates to the question of “what” must be learned. Intrinsic load increases when encountering novel material, and the learner has no prior experience with the topic and does not possess matching schema to process the material. While very high intrinsic load aggravates learning, very low cognitive load triggers boredom and the impression of wasting time (e.g., asking a mathematics student to multiply single-digit numbers Hill and Perkins, 1985; Zakay, 2014). Extraneous load is generated by the means of instruction and refers to the question of “how” the material is presented to the learners (Sweller, 2010; Van Merriënboer and Sweller, 2010). Obviously, when the material is presented in an unorganized, incoherent, and contradicting manner, extraneous load increases and may exacerbate the learning process, for instance due to semantic conflict (Miller et al., 2019). Germane load is generated by the learner’s deliberate use of cognitive resources to use matching schemas to combine information into larger, well-structured, and more retrievable chunks (Sweller, 2010; Van Merriënboer and Sweller, 2010).

According to CLT intrinsic, extraneous, and germane load are additive (Sweller, 2010; Say et al., 2019) and their sum should not exceed the available cognitive resources of the learner. Ideally, extraneous load should be smaller than intrinsic load which, in return, should be smaller than germane load. While intrinsic and germane load are entirely under learners’ control and managed internally, extraneous load is imposed externally and can be altered by means of instructional design techniques (Sewell et al., 2017).

## Expanding and Unpacking the Complexity of the Concept of Extraneous Load

Recently, Mohiyeddini (Taylor et al., 2022) suggested to expand the traditional understanding of the extraneous load to consider the psychological environment in which learning happens (Mohiyeddini, submitted). According to this fundamental fact, “Learning and instructional procedures do not occur in a situational vacuum.” This argument expands the definition of extraneous cognitive load as it assumes that situational characteristics may ease or deteriorate the process of delivery of information and the development, modification, and automatization of cognitive schemas over and above the quality of the instructional process. Therefore, Mohiyeddini (submitted) has suggested that “our concept of ‘extraneous cognitive load’ must encompass the broad diversity and complexity of situational factors under which learning materials are presented.” Situations, such as the COVID-19 pandemic, are highly ambiguous and suggest a serious threat for individual’s survival. Therefore, we must consider the impact of COVID-19 in causing fear, anxiety, and triggering worrisome thoughts (Castelnuovo et al., 2020).

## Alleviating Extraneous Load for Instructors

As already highlighted, extraneous load is generated by the means of instruction and it is not essential to comprehending the information. However, it occupies cognitive resources and may overwhelm the WM. Building upon our recent work (Sewell et al., 2017; Jordan et al., 2020), we endeavored to lessen the extraneous load for departmental faculty in shifting toward online-only teaching. We utilized and adapted the following well-researched principles of CLT (Van Merriënboer and Sweller, 2010).

*Split attention principle: this principle suggests providing one integrated source of information, avoiding temporal split attention (sources distributed in time), and/or spatial split attention (sources distributed in space).*

We anticipated that two different clusters of information produced by the COVID-19 environment might overwhelm the faculty’s attention, challenge the faculty’s locus on control (Sigurvinsdottir et al., 2020), and create confusion (Partlak Günüşen et al., 2014; Seixas et al., 2015): *Firstly*, information related to how to create a functioning and efficient home office environment suitable for online activities, such as teaching, supervision, mentoring, and publishing. To manage and integrate this information and enhance the faculty’s sense of control, at the beginning of the crisis we established usage of a department-wide team communication platform (Slack^R^).^1^ This step is supported by empirical findings that show access to valid and reliable information reduces ambiguity and anxiety (Stamenkovic et al., 2018; Sancak and Akal, 2019). We used our departmental Slack workspace in order to integrate faculty’s briefing, collaborations, and conversations into one tool—ideal for newly dispersed work schedules. *Secondly*, we sought to manage COVID-19-related health and safety information. However, this has been particularly difficult to address, with separate, often disparate, and inconsistent information coming from the federal and state officials, the Centers for Disease Control, and our clinical partner, to name a few. Therefore, departmental administration has committed itself to “staying up” with current information and providing that information with high-fidelity to the faculty. To deal with this challenge, and as a supplement to our daily “water cooler” conversations, we developed a “COVID-19 updates” Slack channel (channels are separate discussion spaces for sub-groups/specific topics) to share information updates both from within our own health system as well as national/scientific news. This channel offers the faculty the most up-to-date scientific news as collated and vetted by colleagues. By extension, this channel was used to scientifically inform relatives, friends, and communities of our faculty and to reduce fear and confusion among them.

*Goal-free principle: this strategy suggests substituting conventional tasks with goal-free tasks that provide learners with a non-specific goal.*

One of our first efforts in addressing potential issues for the faculty was to ask, both individually and collectively, “what do you need from us” to successfully continue with your teaching and other scholarly activities. This strategy not only provided us with a list of items to be addressed but had the effect of reducing their extraneous stress by allowing them to put anything they wanted “on the table.” Outcomes from this effort included: (1) a long list of (mostly) technology needs for solving both teaching and faculty office-related issues—such that productivity could be maintained and in some cases even increased during the period of remote working; (2) software and in some cases hardware updates and upgrades; and (3) flexibility with work from home schedules.

*Worked example principle: this strategy suggests providing learners with worked examples that offer a full solution that learners must cautiously study and comprehend.*

During our orientation to introduce Slack as a communication tool, we were fortunate to have already piloted its use in a course within our department, thus two of the authors of this manuscript already had familiarity with its use. As the Slack tool was new for most department members, we immediately provided a basic “how to” document. We also quickly scheduled a departmental workshop with the tool’s implementation, just prior to the work from home order. Additionally, in the early days of departmental Slack usage, the authors sought to create example communications within the platform to demonstrate its benefits. For example, an individual enquiry regarding how to voice record over a lecture slide was posted in our online teaching resources channel and led to eight separate faculty members offering guides and helpful tips. These resources not only led to a great dialogue of shared experiences, but also lives as a searchable resource for a future similar question. Further, we offered faculty members several faculty developments workshops for online course delivery. These experiences were delivered by two expert departmental faculty members in online teaching who were able to reduce many of the unknowns for the faculty by providing detailed worked examples of how to successfully deliver online teaching.

*Completion principle*: this principle suggests providing *a partial solution a learner must finish instead of the traditional task.*

We demonstrated how to setup channels in Slack by creating individual discussion spaces (channels) for all departmental Scholarly Interest Groups (SIGs). Departmental SIGs are designed to mirror more traditional laboratory group meetings to increase peer-accountability, scholarly output, and rigor and constructive criticism. We also initially created channels for discussions pertaining to “Online Teaching Resources,” “Instructional Design,” “Medical Library,” and “M1/M2 Curriculum.” Following these demonstrations, faculty were assisted to create additional collaborative channels to encourage continued dialogue and pursuit of ongoing scholarly projects. In just over one month of usage, and as supporting evidence for our approach to reduce the extraneous cognitive load for our faculty, our department sent close to 10,000 messages and created over 20 collaborative channels and message groups within the first month of implementation. Not surprisingly, most Slack usage occurs during the normal business day, but faculty have noted that the platform is also useful when collaborating with colleagues whose schedules have become more dispersed due to working from home.

*Modality principle: this principle suggests providing a narrated text to a visual source (multimodality) instead of combining the visual source with an explanatory text in writing (unimodal).*

We solicited detailed guidelines from the faculty to develop digital guides for online teaching practices. Furthermore, our faculty created narrated demonstrations of available tools in best practices in online teaching. Such efforts were complimented by main campus resources, which were identified and vetted, including: workshops, guides, lectures, and self-paced online courses all to develop online presentation and course management skills.

*Redundancy principle: Replace multiple self-contained sources with one source of information.*

We investigated all available technology options already in place within our curriculum (including Moodle curriculum management system tools, WebEx, USeeYou, Google Hangouts, and Panopto lecture recording). We created one-page “how-to” guides for less frequently used software tools, and have excelled in the curation of stand-alone and vetted resources for the faculty to use (specifically pointing to previously little-used tools existing within Moodle and Google suite for active learning and easy to schedule collaborative online meetings respectively). Such resources have been provided in Slack, email, and in online presentations. If a faculty member has the motivation to learn more about flourishing in a virtual world—the information is at their fingertips.

## Supplemental Strategies: Using Humor to Defuse Anxiety and Pressure

Past research suggests that humor can defuse anxiety, reduce worrisome thoughts and psychological pressure on individuals facing uncertain times (Savage et al., 2017; Fang et al., 2019; Menéndez-Aller et al., 2020). The COVID-19 pandemic forced social isolation upon society, harboring the potential danger of creating physical and psychological isolation from other coworkers and students. As a department used to operating in a collaborative space with much face-to-face interaction, we knew that the move to online could be distinctly disruptive to our typical work environment. To keep our department’s collegiality and sense of normalcy alive-and-well while we operate online, we created a “Comic Relief” channel and have had a daily “welcome to work” morning group message.

## Discussion and Conclusion

A recent systematic review of 39 articles shows that many medical schools successfully implemented a variety of web-based resources and developed novel interactive forms of virtual teaching (Wilcha, 2020). However, our analysis of these articles and our database searches in PubMed, Eric, PsycINFO, and PsycARTICLES revealed that our paper seems to be the first that demonstrates the applicability of Cognitive Load Theory as a traditional cognitive theory of learning in the field of academic management to provide feasible strategies to ease the cognitive loads of instructors.

This paper adds to our knowledge on CLT, online teaching, and virtual collaborations in several unique ways: First, this paper demonstrates that psychological theories are useful and applicable to guide the process of change; they are even applicable to guide sudden change, change that is not the consequence of deliberations nor advanced planning, even change forced upon an academic institution. The rationale provided in this paper could be used to develop, maintain, and enhance online teaching, supervision, and research activities during normal times considering the digital nature of our socially distant world. This approach could also be used to proactively develop contingency plans and toolboxes to maintain high-quality teaching in dealing with national and international disasters in the future. Second, while we fully acknowledge the negative impacts of the COVID-19 pandemic on our lives, societies, and international relations, we moved toward a strength and resources-focused approach and adapted a positive psychological view of the COVID-19 situation to enhance our online teaching and scholarly capabilities and activities. We shifted the focus of CLT toward instructors/faculty members, demonstrating that CLT is applicable and beneficial in terms of faculty development and must be similarly addressed. Thirdly, our adaptation of CLT principles to reduce extraneous load, such as the split attention principle, indicates that these principles may serve to enhance the psychological environment for all learners by reducing fear and worrisome thoughts because of exposure to scientifically questionable information and suggestions. We believe that our sudden, yet organized move toward an online teaching and work environment, enhanced the perception of the internal locus on control among faculty (Partlak Günüşen et al., 2014) by enhancing the sense of personal control over the situation. Our approach also highlights that academic management can leverage educational and psychological theories on learning to provide theory-based guidelines to faculty. Furthermore, we believe that holding the immediate in person workshop to demonstrate Slack decreased the sense of helplessness (Pittman and Pittman, 1979; Shaw, 2020) among faculty by ensuring that all faculty were at least set up in the platform just prior to campus closing.

Third, our approach delivers an encouraging example of how to create a virtual collaborative space for scholarly activities which adds to traditional face-to-face scholarly skills and resources. As already highlighted, our approach can foster the quality of teaching and research during normal times. Cultivating a culture of internal online collaborations can be beneficial to maintaining and fostering national and international collaborations as well.

Although the aim of this paper was to shift the focus of CLT toward instructors, our theory-based approach may also be beneficial for our students. Relying on transactional theories of stress (e.g., Lazarus, 1966; Lazarus and Folkman, 1984) we assume that reducing instructors’ stress by reducing their cognitive overload will, by extension, benefit the students. Previous research has demonstrated that instructors play a key role in mitigating students’ stress to progress learning (Willis et al., 2021).

The main limitation of this paper, as is inherent to the nature of an opinion piece, is the lack of data to offer empirical evidence to support our claims and suggestions. Furthermore, it is based on an individual academic institution’s actions and, hence, lacks external validity. However, looking to the future with the potential for continued remote teaching, and by using theory-driven learning strategies, we aim to provide empirical data to highlight the strength and shortcomings of our approach to minimize the cognitive load for faculty required to succeed in all aspects of their career.

## Author Contributions

CM designed this paper. DG, KS, and CM wrote the paper together and contributed to all sections of the paper. All authors contributed to the article and approved the submitted version.

## Conflict of Interest

The authors declare that the research was conducted in the absence of any commercial or financial relationships that could be construed as a potential conflict of interest.

## Publisher’s Note

All claims expressed in this article are solely those of the authors and do not necessarily represent those of their affiliated organizations, or those of the publisher, the editors and the reviewers. Any product that may be evaluated in this article, or claim that may be made by its manufacturer, is not guaranteed or endorsed by the publisher.

## Footnotes

^1^ www.slack.com
